# Palm Oil-Free Structured Lipids: A Novel Structuring Fat for Sandwich Cookie Fillings

**DOI:** 10.3390/foods15010178

**Published:** 2026-01-05

**Authors:** Vanessa Alves, Guilherme de Figueiredo Furtado, Matheus Augusto Silva Roman, Lígia de Meyer Pacheco Delboni, Juliana Alves Macedo, Carla Lea de Camargo Vianna, Valdecir Luccas, Gabriela Alves Macedo

**Affiliations:** 1 Food Science and Nutrition Department, School of Food Engineering, Universidade Estadual de Campinas (UNICAMP), Monteiro Lobato, 80, Campinas 13083-862, SP, Brazil; alvanessa92@gmail.com (V.A.); sts.matheus@gmail.com (M.A.S.R.); ligiadelboni20@gmail.com (L.d.M.P.D.); jumacedo@unicamp.br (J.A.M.); 2 Centre of Natural Sciences, Federal University of São Carlos (UFSCar), Rod. Lauri Simões de Barros, Km 12–SP 189, Buri 18290-000, SP, Brazil; furtado.gf@gmail.com; 3 Institute of Food Technology (ITAL), Cereal and Chocolate Technology Center, Avenida Brasil, 2880, Campinas 13070-178, SP, Brazil; carla.lea@ital.sp.gov.br (C.L.d.C.V.); vluccas@ital.sp.gov.br (V.L.)

**Keywords:** hard fat, behenic acid, enzymatic interesterification, strawberry filling

## Abstract

This study aimed to evaluate the efficacy of a palm oil-free structured lipid (SL) as a lipid base in sandwich-type cookie fillings. SL was enzymatically interesterified from a blend of soybean oil, high-oleic peanut oil, and crambe hard fat (34:34:32). Four fillings (30% fat) were prepared using either SL or commercial shortening (CS), with processing by mixer (F1, F2) or ball mill (F3, F4). Commercial sandwich cookies were included as a reference Standard. SL exhibited an improved lipid profile, containing up to 14% less saturated fatty acids, higher levels of monounsaturated (34.5%), and increased long-chain fatty acids (~18% C22:0). Physicochemical analyses were conducted over a storage period of 180 days, including evaluations of texture, particle distribution, color, water activity, oil loss, and oxidative stability. Among the formulations, F4 demonstrated the highest firmness (at ~121.1 N) and the smallest D_50_ (~80 µm). However, it also exhibited lower oxidative stability (induction period: ~6.75 to 14.6 h) compared to CS-based fillings (~36 to 42.5 h), along with a higher oil loss (≥4.7%). Sensory of F4 yielded an overall acceptance index ≥ 70%, though it received lower scores for waxiness. Overall, the SL showed promising potential as a structuring fat in the fillings.

## 1. Introduction

Sandwich cookies consist of two cookie or wafer layers with an intermediate filling, forming a “sandwich” structure [1]. Fillings typically contain 30–35% fat and sugar, with consistency determined by solid fat content, and may include starch, emulsifiers, flavors, and colorants [2,3].

Fat is essential in fillings, providing the structural matrix that dictates texture, stability, and overall sensory quality of the final product. However, it is well established that high intake of saturated fatty acids is a risk factor for the development of obesity and may contribute to the onset or worsening of metabolic disorders and conditions associated with non-communicable chronic diseases (NCDs) [4]. In this context, health authorities and regulatory agencies involved in nutrition and public health are at various stages of evaluating and implementing policies to promote healthy dietary habits, favoring vegetable oils rich in mono and polyunsaturated fatty acids (MUFAs and PUFAs) and reducing the intake of major sources of saturated fats to less than 10% of total energy intake [5].

Currently, fats commonly used in cookie fillings include coconut oil, palm kernel oil, palm oil, and their fully hydrogenated fractions blended with vegetable oils rich in unsaturated fatty acids [3,6]. However, the consumption of palm fat has been questioned due to saturated fatty acid contents ≥ 50 wt%, and its use has been widely debated because of growing concerns about potential environmental and social impacts associated with the expansion of cultivation areas, particularly in Southeast Asia [7]. From a technological perspective, excessive amounts of undesired by-products such as diacylglycerols (DAGs) and contaminants including 3-monochloropropane-1,2-diol (3-MCPD) esters and glycidyl esters can be generated during fractionation and refining processes [8]. Other approaches, including fat replacement with carbohydrates or the use of oleogels and hydrocolloids, have shown promise but may introduce nutritional or functional trade-offs [9,10,11,12,13].

However, palm oil’s high saturated fat content, alongside environmental concerns regarding its cultivation and the potential formation of processing contaminants, has driven the search for alternatives [14,15,16]. The structured lipids (SLs) can be obtained through enzymatic or chemical interesterification, a viable and promising method for the production of dietary lipids, allowing the blending of hard fats and low-cost vegetable oils rich in essential and/or nutritionally relevant fatty acids (low-calorie) [17].

In previous studies conducted by our research group, Alves et al. [18] produced SLs through chemical and enzymatic interesterification, palm oil–free, using a blend of soybean oil and high-oleic peanut oil with the gradual addition of crambe oil hard fat to increase the C22:0 content. This resulted in a new palm-oil–free fat portfolio, associated with the patent application BR1020240090128, filed with the National Institute of Industrial Property (INPI), Brazil [19]. Both interesterification processes were effective in lipid modification; however, enzymatic SLs were preferred because they are more eco-friendly and exhibit greater plastic fat characteristics suitable for diverse food applications (e.g., margarine, spreads, fillings, coatings). Since there are no reports in the literature specifically on the application of palm oil–free SLs as a lipid base for cookie fillings, we selected one of the enzymatic SLs, containing approximately 18% C22:0, for this application due to its technological functionality as a structuring fat, which is comparable to commercial shortening used in fillings. Therefore, this study aimed to reproduce the enzymatic SL, rich in MUFAs and C22:0, and apply it as a total replacement for palm-based shortening in sandwich cookie fillings, producing palm oil–free fillings as a healthier and more sustainable alternative.

## 2. Materials and Methods

### 2.1. Materials

The materials used for the preparation of the SL were: soybean oil (SO) (Liza, Cargill Incorporated, Uberlandia, MG, Brazil), purchased from a local market in Campinas, SP, Brazil; high-oleic peanut oil (PO) (Sementes Esperança Comércio, Importação e Exportação Ltda., Jaboticabal, SP, Brazil); and fully hydrogenated crambe oil hard fat (HFCO) (Chemyunion Ltda., Sorocaba, SP, Brazil). For the enzymatic interesterification reaction, commercial immobilized lipase from *Thermomyces lanuginosus* (Lipozyme TL IM, Novozymes Latin America Ltda., Araucaria, PR, Brazil) was used. According to the manufacturer, the biocatalyst exhibits an enzymatic activity of ~250 IUN g^−1^, shows *sn*-1,3 positional specificity toward triglycerides, and operates optimally within a temperature range of 50–75 °C, with a moisture content ≤8.0% (loss on drying at 105 °C). These parameters were used to define the reaction conditions and enzyme loading in this study.

For the production of the fillings, the following materials were used: granulated sugar and powdered sugar without starch (Mais Doce, Açucareira Boa Vista, Limeira, SP, Brazil), shortening Vivali 35E LTS NP (Bunge Alimentos S.A., Gaspar, SC, Brazil), soy lecithin (Solec^TM^—International Flavours & Fragrances—IFF, Gibson City, IL, USA), red vegetable concentrate (colorant) (Exberry^®^, GNT, Sao Paulo, SP, Brazil), natural strawberry flavor (Solutaste—Soluções & Sabores, Diadema, SP, Brazil), and commercially available strawberry-flavored sandwich cookies were obtained from Marilan Alimentos S.A., produced at the company’s Northeastern manufacturing unit (Igarassu, PE, Brazil).

### 2.2. Development of the Structuring Lipid

The SL was prepared according to the methodology of [18], using a 1:1 (wt%) blend of SO and PO with the addition of 32 wt% HFCO. The oil blend was fully melted at 100 °C for 10 min to erase the crystallization memory, under continuous stirring to ensure complete homogenization [20]. Enzymatic interesterification was conducted at 60 °C for 6 h with 5 wt% Lipozyme TL IM under vacuum and stirring. The lipase was removed by filtration.

### 2.3. Characterization of Fats

#### 2.3.1. Fatty Acid Composition (FAC)

The FAC was analyzed by gas chromatography–mass spectrometry (GC–MS QP2010S, Shimadzu, Kyoto, Japan) after esterification according to [21]. Fatty acid methyl esters were separated following Ce 1f-96 [22], using a DB-23 capillary column (60 m × 0.25 mm × 0.25 µm, Agilent). Helium was used as carrier gas (1.0 mL/min). The injector and detector were set at 250 and 280 °C, respectively. The oven was programmed from 110 to 215 °C at 5 °C/min, with a 24 min hold at 215 °C. A 1.0 µL sample was injected in split mode (1:50). Fatty acids were identified by comparing retention times with authentic standards.

#### 2.3.2. Triacylglycerol (TAG) Composition

The TAG composition was analyzed according to Ce 5-86 [22], using gas chromatography (GC Agilent 6850, Santa Clara, CA, USA) equipped with a DB-17 HT capillary column (15 m × 0.25 mm × 0.15 µm, Agilent). Helium was used as carrier gas (1.0 mL/min). The injector and detector were set at 360 and 375 °C, respectively. The oven was programmed from 280 to 340 °C at 2 °C/min and held at 340 °C for 40 min. The samples (10 mg/mL in THF) were kept at room temperature (~21 ± 2 °C) and injected immediately after preparation in split mode (1:100). TAGs were identified using PrOleos^®^ software [23], available online through the LAMES platform (Laboratory of Extraction and Separation Methods, Federal University of Goiás, Brazil; https://lames.quimica.ufg.br/p/4035-courseware, accessed on 13 March 2025), by predictive modeling and comparison of retention times with probable TAGs of higher percentage within groups of given carbon numbers (CN).

#### 2.3.3. Solid Fat Content (SFC)

The SFC was analyzed by nuclear magnetic resonance spectrometry (NMR, Bruker pc120 Minispec, Germany) coupled with dry baths operating from 0 to 70 °C (Tcon 2000, Duratech, Noblesville, IN, USA) according to Cd 16b-93 [22]. Samples were melted at 60 °C (5 min), stabilized at 0 °C (60 min), and measured at 10–50 °C (increments of 5–10 °C) every 30 min until complete melting.

#### 2.3.4. Thermal Behavior

Crystallization and melting thermal profiles were analyzed by differential scanning calorimetry (DSC), model Q250 (TA Instruments, New Castle, DE, USA), according to Cj 1–94 [22]. Lipid samples (8–10 mg) were weighed into aluminum pans, with an empty hermetically sealed pan as reference. The analysis conditions were as follows: samples were held at 80 °C for 10 min and then cooled at a rate of 10 °C/min to −80 °C. After this cycle, samples were heated from −80 °C to 80 °C at a rate of 5 °C/min. Thermal curves were recorded using TRIOS software, version 5.1.1 (TA Instruments, New Castle, DE, USA), from which the onset temperature of crystallization (T_onset_), peak crystallization temperature (T_peak_), end crystallization temperature (T_fc_), and crystallization and melting enthalpies (ΔH) were determined.

### 2.4. Preparation of Cookie Fillings

To produce the fillings, the commercial shortening (CS) was used as the control and was completely replaced by the SL at 30 wt% lipid base, based on the average composition of commercial fillings [3].

The fillings were prepared using two methods: a stand mixer and a ball mill. The stand mixer reproduces, at pilot scale, the industrial creaming process, ensuring homogeneous mixing, ingredient dispersion, and proper aeration. The ball mill simulates the refining and homogenization stage, providing comparable particle size and texture to large-scale production. Comparing both methods verifies the ability of pilot-scale processes to replicate industrial conditions while allowing formulation adjustments with lower material use and precise control of time, speed, and temperature, ensuring reproducibility and product quality.

**Figure 1:**
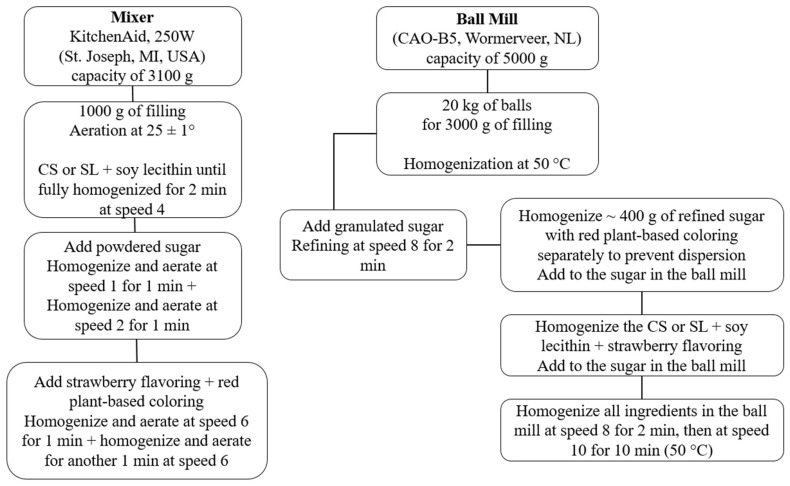
Steps in the production of fillings for sandwich-type cookies.

Four filling formulations were developed: F1, containing 100% CS, and F2, containing 100% SL, both produced using the mixer method; and F3, formulated with 100% CS, and F4, with 100% SL, both processed using the ball mill method. As a reference, commercially available sandwich cookies were also included and referred to as Standard (S). The fillings were prepared at the pilot plant of Cereal Chocotec (Institute of Food Technology, Campinas, Brazil). The production steps of the fillings are described below, as illustrated in Figure 1.

#### Sandwich Cookie Assembly

The fillings obtained using the mixer and ball mill methods were applied onto the inner surface of cookie shells using a 60 mL graduated syringe (Luer-Lok), with a volume of 4 mL per cookie (~3 g), followed by placement of the second cookie shell, forming a sandwich. The assembled sandwich cookies were then conveyed through an 8 m cooling tunnel at 5 °C, with an average residence time of 13.5 min and a belt speed of approximately 20 m/h, to allow lipid crystallization of the fillings. Subsequently, groups of ten cookies were packed in metallized biaxially oriented polypropylene (BOPP) film and stored at 23 ± 1 °C until further analyses.

### 2.5. Characterization of Strawberry Fillings

The particle size distribution (PSD) was determined by laser diffraction (LA-950, HORIBA, Ltd., Kyoto, Japan) according to ISO 13320 [24]. Approximately 3 g of each filling sample was dispersed in an ethanol solution, employing the instrument’s integrated circulation and ultrasonic system to ensure proper homogenization. For optical parameters, the refractive index was set to 1.45 and the absorption index to 0.1. Results are reported as volume-weighted mean diameter (D _[4.3]_), median diameter (D_50_), and distribution width (Span). Surface color was measured with a colorimeter (CR-10, Konica Minolta, Inc., Tokyo, Japan) in the CIE L*a*b* system, reporting L, a, b, chroma (C), and hue angle (h°), following the ISO 11664-4 method [25]. The water activity (*a*_w_) was measured at 25 °C using a water activity meter (Decagon AquaLab Devices, Inc., Pullman, WA, USA), following the AOAC 978.18 method [26]. The fillings were carefully removed from the shells and analyzed separately, while the shells were ground into a fine powder to ensure sample homogeneity, using approximately 1 g of material.

#### 2.5.1. Texture of Fillings

Texture parameters of the fillings, including firmness, consistency (maximum peak force, N, and area under the curve, N•s), and adhesiveness (negative peak force, N), were evaluated using a TA-XT Plus texture analyzer (Stable Micro Systems Ltd., Godalming, UK; load cell 5 kgf [50 N]) via a penetration test with a 45° acrylic cone probe (3 mm diameter) at 25 ± 1 °C. Test conditions were: initial distance 10 mm/s, test speed 2 mm/s, and contact time 1 s, following [27]. Data were processed using Texture Expert Connect version 8.0 (Stable Micro Systems^®^, Brazil).

#### 2.5.2. Fracturability Test of Cookie Shells

Cookie shell fracture was measured using a TA-XT Plus texture analyzer (Stable Micro Systems Ltd., Godalming, UK; load cell 5 kgf [50 N]) equipped with a three-point bending rig (HDP/3PB) with a 20 mm support span. For the analysis, fillings were carefully removed from the shells, which were tested independently. The procedure followed by [28] with a pre-test speed of 1.0 mm/s, a test speed of 2.0 mm/s, a post-test speed of 10 mm/s, a compression distance of 10 mm, and a trigger force of 0.25 N (~25 g). The maximum force at the first peak was recorded as fracture force (N) and the corresponding position as fracture distance (mm). Force and distance calibrations of the instrument were conducted before testing.

#### 2.5.3. Determination of Oil Loss

Oil loss was evaluated using the gravimetric method on filter paper as described by [29]. Approximately 3 g of filling was placed on circular filter papers (Whatman Nº. 4) and set in Petri dishes. The fillings remained in continuous contact with the filter papers throughout the storage period and were removed only at each evaluation time to determine oil absorption. Oil loss measurements were performed after 7 days of storage and subsequently at 30-day intervals until completing 180 days of analysis at 25 °C. Oil loss was calculated according to Equation (1).

Oil loss (%) = (m_ff_ − m_if_)/m_sample_ × 100% (1)

where m_if_ is the initial mass of the dry filter paper (g), m_ff_ is the mass of the filter paper after oil absorption (g), and m_sample_ is the initial mass of the filling placed on the filter paper (g).

#### 2.5.4. Oxidative Stability

The lipid fraction of the fillings was extracted by homogenizing the samples in petroleum ether, followed by an overnight period to promote phase separation. The upper solvent phase was carefully collected and filtered through filter paper containing anhydrous sodium sulfate to remove residual moisture. The filtrate was subjected to solvent evaporation in a rotary evaporator under vacuum at 60 °C until complete removal of the ether. The oil obtained was flushed with nitrogen for 15 min to prevent oxidation, and the lipid fraction was then weighed. Oxidative stability of the extracted oil was assessed using a Rancimat apparatus (Metrohm 743, Herisau, Switzerland), following the Cd 12b-92 method [30], with 3.5 to 5.0 g of sample at a constant temperature of 100 °C and continuous air flow of 10 L/h. Results were expressed in hours, corresponding to the induction period of lipid oxidation.

### 2.6. Sensory Analysis

Strawberry-flavored sandwich cookies were evaluated for acceptance and purchase intention by 65 untrained panelists (mean age 34 years). Samples were coded and served on polyethylene plates with mineral water for palate cleansing. Sensory attributes assessed were: aroma, flavor, color, grittiness, waxiness, crunchiness, and overall acceptance, using a nine-point hedonic scale, where: 1 = “disliked extremely”, 2 = “disliked very much”, 3 = “disliked moderately”, 4 = “disliked slightly”, 5 = “neither liked nor disliked”, 6 = “liked slightly”, 7 = “liked moderately”, 8 = “liked very much”, and 9 = “liked extremely”. Purchase intention was measured on a five-point scale (1 = “certainly would not purchase” to 5 = “certainly would purchase”) following [31,32]. The study was conducted at the Sensory Laboratory of the Institute of Food Technology, Campinas, Brazil, and approved by the Research Ethics Committee (CAAE: 80457424.2.0000.5632), and informed consent was obtained from each subject before they participated in the study. Volunteers regularly consumed sandwich cookies; individuals with diabetes or peanut allergies were excluded.

### 2.7. Statistical Analysis

Statistical analysis of the data was performed to determine significant differences among means at a 5% probability level (*p* < 0.05) using one-way ANOVA followed by Tukey’s test. Pearson correlation coefficients were also calculated to assess associations between variables. Analyses were conducted using Minitab for Windows, version 16.2.2.

## 3. Results and Discussion

### 3.1. Characterization of Lipid Bases

#### 3.1.1. Fatty Acid Composition and Triacylglycerol Profile

Table 1 shows the comparison of fatty acid compositions between SL and CS.

Fatty acid profile (Table 1) revealed the defining characteristics of the SL. Compared to the CS, the SL contained significantly less palmitic acid (7.32% vs. 44.10%) but higher levels of stearic (10.77% vs. 6.13%) and oleic acid (34.50% vs. 23.94%). Most notably, the SL was enriched with ~18% behenic acid (C22:0), which was absent in CS. Consequently, the total saturated fatty acid content was reduced by approximately 14% in the SL (38.92%) compared to CS (52.46%).

This saturated fatty acid composition in the SL may be highly effective in improving the nutritional properties of cookie fillings, as its total replacement could provide lower-calorie fillings due to the high content of long-chain fatty acids, such as behenic acid (~18%), which may inhibit pancreatic lipase activity, resulting in an anti-obesogenic effect due to partial absorption by the body, as reported by [33,34,35,36]. Batista et al. [37] reported that enzymatic interesterification is an effective method for producing SLs with lower caloric content, observing that this process led to a 10.6% reduction in the in vitro digestibility of SLs compared to the native oil.

Regarding TAG content, the SL exhibited a reduction in S_3_ (tri-saturated) and S_2_U (mono–and di-saturated) species, associated with hard fat, and an increase in SU_2_ (mono–mono-di-unsaturated) and U_3_ (tri-unsaturated) species, corresponding to the unsaturated fraction of its lipid composition (Table 1). A complete and detailed description of the FAC and TAG corresponding to the SL is provided in [18]. The main TAGs in the SL were OLL (oleic–linoleic–linoleic), LLL (linoleic–linoleic–linoleic), and StLBe (stearic–linoleic–behenic), with the formation of new long-chain TAGs such as BeBeBe (behenic–behenic–behenic) in NC 66. For the CS, TAGs were mainly composed of S_2_U and SU_2_, accounting for 74.91% of its total TAG content. The main TAGs were POP (palmitic–oleic–palmitic), PLP (palmitic–linoleic–palmitic), POO (palmitic–oleic–oleic), PLO (palmitic–linoleic–oleic), and PLL (palmitic–linoleic–linoleic), with ~8% PPP (palmitic–palmitic–palmitic), corresponding primarily to palmitic acid.

#### 3.1.2. Solid Fat Content Profile

**Figure 2:**
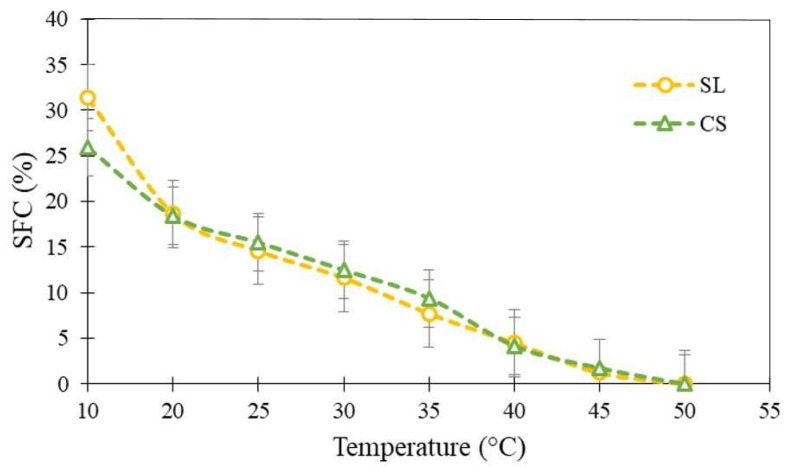
Solid fat content of the commercial shortening (CS) and structured lipid (SL).

Figure 2 shows the comparison of SFC between the CS, based on modified palm fat, and the developed SL. The SFC profiles were similar, particularly above 20 °C, with a melting point around 50 °C, although the SL exhibited a slightly higher solid content (31.37%) than the CS (25.91%) at 10 °C. At 25 °C, the SFC of the SL is approximately 20%, which is consistent with the requirements for its application as a lipid base in cookie fillings, according to [6,38], as it meets the specifications for spreadable products. The SFC is considered adequate for products resistant to deformation, ensuring that fillings maintain their structure when mechanically deposited onto cookie shells, retaining the sandwich form. According to [39], this increase in SFC in the SL can be attributed to the high concentration of behenic acid (~18%) from HFCO, which has a long chain and a high melting point (≥65 °C), promoting the formation of a structured and stable crystalline network, resulting in SFC with improved consistency, firmness, and stability in bakery products and fillings.

#### 3.1.3. Thermal Profiles of Crystallization and Melting Point

**Figure 3:**
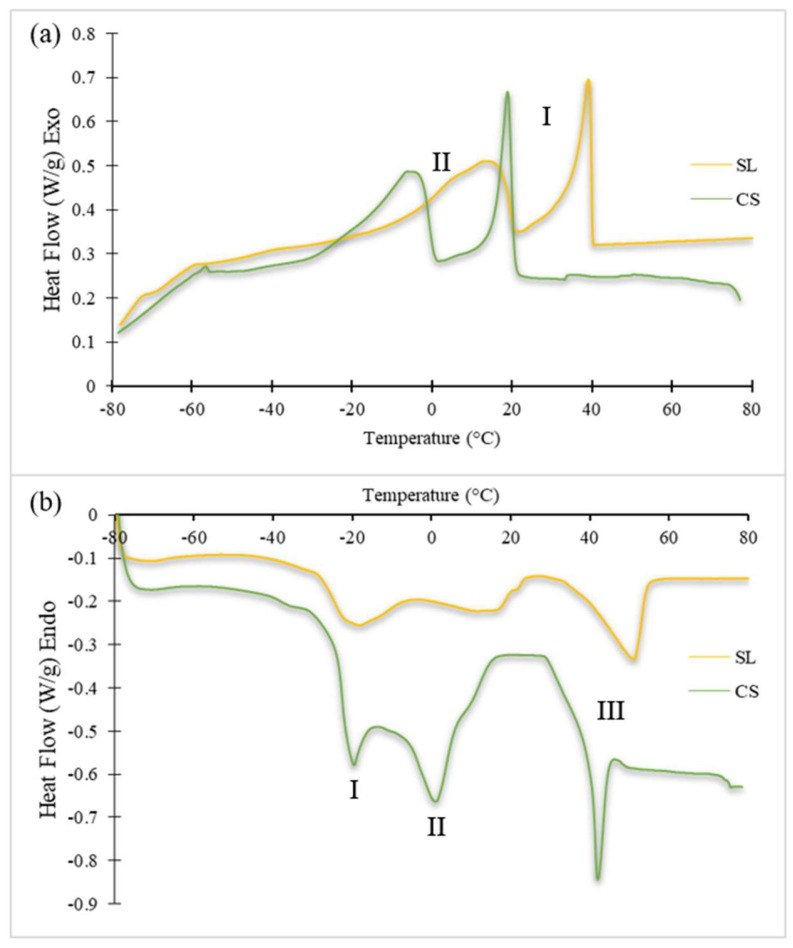
Thermograms: (**a**) Crystallization; (**b**) melting.

The crystallization profile revealed two exothermic events for both SL and CS (Figure 3a). In the first event, SL crystallized at higher temperatures, with an onset at 39.73 °C and a peak at 38.99 °C, while CS began at 20.50 °C and peaked at 18.86 °C. The second, broader event occurred at lower temperatures: SL crystallized from 20.46 °C with a peak at 14.53 °C and concluded at −6.53 °C; CS initiated this event at 0.77 °C, peaked at −4.40 °C, and completed crystallization at −26.31 °C. The SL also showed a higher crystallization enthalpy (13.952 J/g) than CS (8.3135 J/g), consistent with the predominance of SU_2_ and U_3_ TAGs, while CS contained more S_2_U. Although both fats exhibited similar melting profiles (Figure 3b), spanning −29.18 °C to 50.17 °C, CS presented more prominent high-melting peaks (around 45–50 °C). The SL, however, required more energy for complete melting, with a melting enthalpy of 23.920 J/g, compared with 17.157 J/g for CS.

According to [40] the TAG and FAC of fats and their blends directly affect crystallization behavior, influencing onset temperatures and peak shifts. These findings align with [38] who reported three melting peaks in interesterified blends of lard, canola oil, and fish oil concentrate for cookie applications. The presence of long-chain PUFA-rich TAGs broadened the melting range, which the authors noted as desirable for bakery applications.

### 3.2. Characterization of Fillings and Sandwich-Type Cookies

#### 3.2.1. Physicochemical Characterization

The particle size distribution (PSD) was significantly influenced by the production method (Table 2). Fillings processed by the ball mill (F3, F4) had markedly smaller median PSD (D_50_) compared to those from the mixer, with F4 being the finest (~80 µm) and most similar to the commercial Standard.

Over 180 days of storage, a general reduction in PSD was observed across all samples, likely due to matrix reorganization or crystal fragmentation, with F4 showing the most pronounced decrease. According to [41], in lipid systems, polymorphic transformations and recrystallization processes continue during storage as the system seeks a more energetically stable state, leading to changes in crystal size and morphology that affect the PSD over time, which can cause finer crystals to merge or reorganize into more stable forms during extended storage. Processing conditions also play a role. Borriello et al. [42] demonstrated that ball-mill refining of spreads containing pumpkin seed oil-based oleogels with carnauba wax accelerated PSD reduction. Still, carnauba wax acted as an emulsifier, stabilizing small particles and allowing the spread structure to form.

In the present study, the fillings F3 and F4 were processed in the mill using the same ingredient proportions, differing only in the type of fat. The more pronounced PSD reduction in F4 can be attributed to the structured lipids: oleogels or structured lipid matrices are more susceptible to fragmentation and reorganization during milling, whereas palm fat forms a more stable crystalline network that can resist such changes [43]. This indicates that both the composition and the physical properties of the lipid phase can influence the evolution of PSD during storage.

Akamine et al. [44] reported Span values ranging from 3.2 to 7.8 in strawberry fillings of commercial cookies, indicating high polydispersity of solid particles. According to the authors, this wide variation is associated with sugar crystal size and may result in the sensory perception of grittiness.

Color parameters shifted significantly during storage. All fillings exhibited a progressive increase in redness (a*) and chroma (C), indicating color intensification, whereas the Standard filling maintained a lighter and more stable color, as indicated by higher L* values. The hue angle remained within the red-yellow spectrum for all samples (Table 3). These results are consistent with those reported by [45] for strawberry spreads.

**Figure 4:**
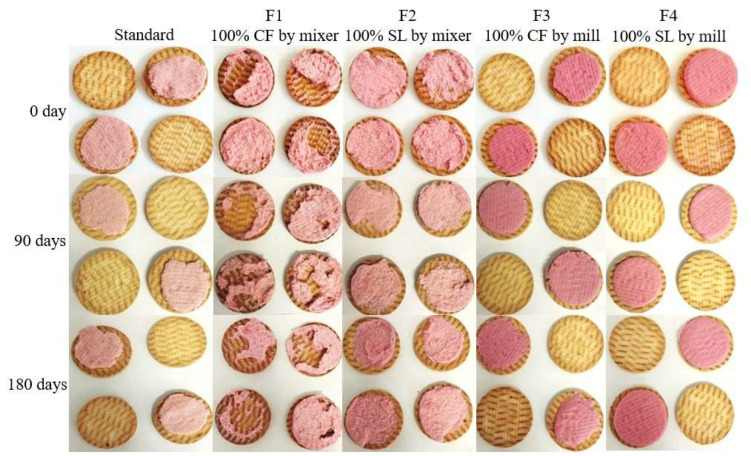
Visual appearance of sandwich-type filled cookies.

The appearance of commercial sandwich-type cookies and those prepared with CS or SL using the mixer and ball mill methods is shown in Figure 4.

Across all fillings, the *a*_w_ initially started at (~0.45–0.58), decreased markedly up to 90 days (~0.36–0.38), and partially recovered by 180 days. The cookie shells the opposite pattern: beginning with lower *a*_w_ (~0.31–0.32) and gradually increasing throughout storage. Moisture uptake was strongest in F1, F3, and F4, while F2 and the Standard remained stable initially before rising later. These complementary trends indicate active moisture migration between fillings and shells over time. The *a*_w_ values of the fillings and their corresponding cookie shells are summarized in Table 4.

The higher *a*_w_ observed in fillings F2 and F4 can be attributed to the use of SLs, which modify lipid–water interactions within the matrix. SL matrices tend to retain more mobile water and create microenvironments where water is less tightly bound, leading to higher *a*_w_ over time. This behavior is consistent with findings by [46], who reported that cookies formulated with structured emulsions exhibited increased water retention and *a*_w_ compared to conventional fat systems, highlighting that the structural organization of the lipid phase strongly influences water availability in composite bakery products.

This behavior suggests moisture migration from the fillings to the cookie shells and/or interaction with environmental humidity, or internal redistribution of water content, the latter being an expected phenomenon in composite products with different initial *a*_w_ values. According to [47] fillings with *a*_w_ ≤ 0.4 are desirable because cookie shells are hygroscopic, and the lack of moisture equilibrium can compromise texture, leading to shell softening, filling hardening, or even separation at the filling:shell interface. Moreover, Sereti et al. [48] highlight that when *a*_w_ exceeds 0.6, microbiological deterioration may occur, affecting the final product quality. The *a*_w_ values of all analyzed fillings ranged from 0.35 to 0.58, remaining below the critical threshold. Pearson’s correlation analysis revealed a strong positive correlation (r = 0.98 for F1, 0.96 for F2, 0.94 for F3, 0.99 for F4, and 0.97 for Standard) between the *a*_w_ values of the fillings and their cookie shells, indicating that as filling *a*_w_ varies, the *a*_w_ of the corresponding shells follows accordingly.

#### 3.2.2. Textural Characteristics

**Figure 5:**
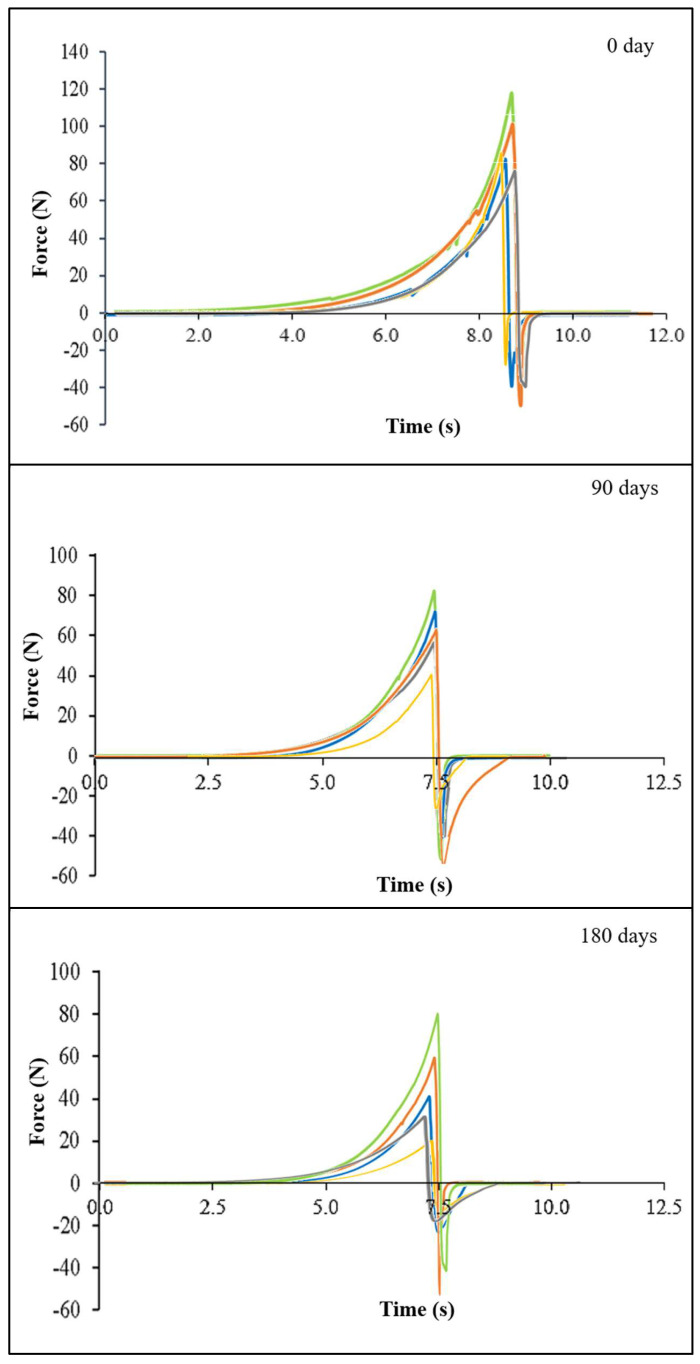
Texture profile of different strawberry fillings. Note: F1 = 100% CS by mixer (■); F2 = 100% SL by mixer (■); F3 = 100% CS by ball mill (■); F4 = 100% SL by ball mill (■), and S = Standard (■).

The textural evolution during storage reflects clear differences in matrix stability among fillings (Figure 5). F1 and F2 softened rapidly, indicating fragile structures that lose integrity as their fat–particle networks reorganize, and moisture redistributes. In contrast, F3, F4, and the Standard maintained greater firmness, with F4 showing the most stable and cohesive matrix, likely due to improved particle dispersion from ball-mill processing. Adhesiveness trends reinforce these mechanisms, and the F1 remains sticky because of poor cohesion, whereas F4 high adhesiveness stems from a stronger, more continuous fat matrix. F2 and F3 become less adhesive as their structures degrade, while the Standard shows controlled, gradual changes typical of industrially stabilized systems, and with TAGs simetrics as PPP (palmitic–palmitic–palmitic) or POP (palmitic–oleic–palmitic). Adhesiveness reflects the filling’s ability to adhere to the cookie, with higher values contributing to the sandwich’s structural integrity [49]. According to [1] an ideal cookie filling should exhibit moderate adhesiveness, avoiding an overly sticky mouthfeel while remaining firm enough to retain its shape and resist collapse during handling or biting.

Furthermore, the differences in texture evolution among fillings during storage can be attributed to TAG symmetry and saturation (Section 3.1.1). Although SL-based fillings contained higher proportions of asymmetric and unsaturated TAGs, e.g., OLL (oleic–linoleic–linoleic) and LLnL (linoleic–linolenic–linoleic), they also included long-chain TAGs such as BeBeBe (behenic–behenic–behenic), which likely increased resistance to melting while still allowing the formation of relatively firm and stable crystalline networks. The higher SU_2_ content favored greater adhesiveness and the development of softer yet more structured matrices, particularly in F4. In addition, ball-mill processing enhanced particle dispersion and lipid phase continuity, thereby amplifying the effects of TAG composition on the overall textural behavior of the fillings.

According to [50,51], more saturated and symmetric TAGs tend to pack more efficiently, forming firmer and more stable crystalline networks. Furthermore, TAGs with longer, saturated fatty acid chains and symmetrical structures are known to favor efficient molecular packing and the formation of stable crystal lattices with higher melting points, resulting in more rigid and firm networks.

**Figure 6:**
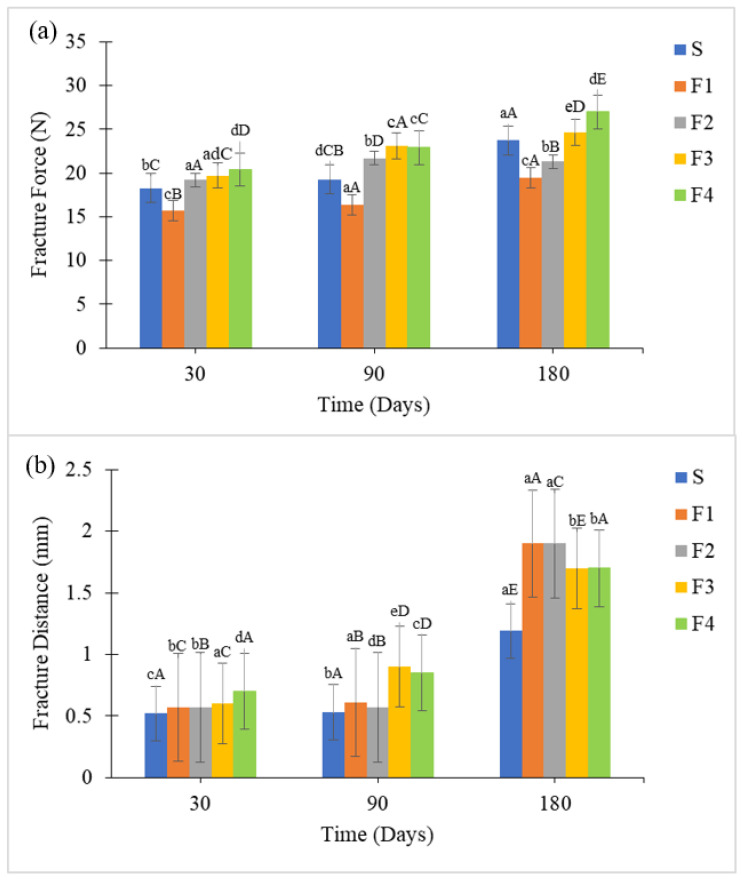
Texture parameters of the cookie shells after 180 days of storage: (**a**) Fracture force (N) and (**b**) Fracture distance (mm). Note: Results marked with different letters showed significant differences (*p* ≤ 0.05). Lowercase letters indicate significant differences between samples, while uppercase letters denote significant differences over time. Formulations: F1 = 100% CS by mixer; F2 = 100% SL by mixer; F3 = 100% CS by ball mill; F4 = 100% SL by ball mill and S = Standard.

The cookie shells showed filling-dependent textural responses during storage (Figure 6). Shells paired with F2, F3, F4, and the Standard developed higher fracture forces over time, suggesting that fillings with more structured internal matrices, whether resulting from ball-mill processing or the use of structuring lipids, contributed to increased shell rigidity through moisture redistribution and matrix interactions. In contrast, shells paired with F1 exhibited the lowest fracture force and shortest fracture distance up to 120 days, indicating higher initial crispness compared to the other fillings. This behavior can be associated with lower oil loss (up to ~4.7%, Section 3.2.3), as the mixer processing promoted less particle breakdown, reducing oil:matrix interactions, while aeration likely slowed oil migration. Across all samples, fracture distance increased throughout storage, indicating a gradual loss of crispness, particularly after mid-storage (around 90 days). This pattern reflects typical processes in filled cookies, where moisture migration, ingredient interactions, and structural relaxation progressively reduce the crisp texture. Filled cookies typically exhibit a long shelf life; however, their textural properties may change during storage due to factors such as moisture migration and redistribution, ingredient interactions, and processing conditions. Among these changes, the most critical quality loss is the reduction in crispness [52].

#### 3.2.3. Oil Loss and Oxidative Stability of Fillings

**Figure 7:**
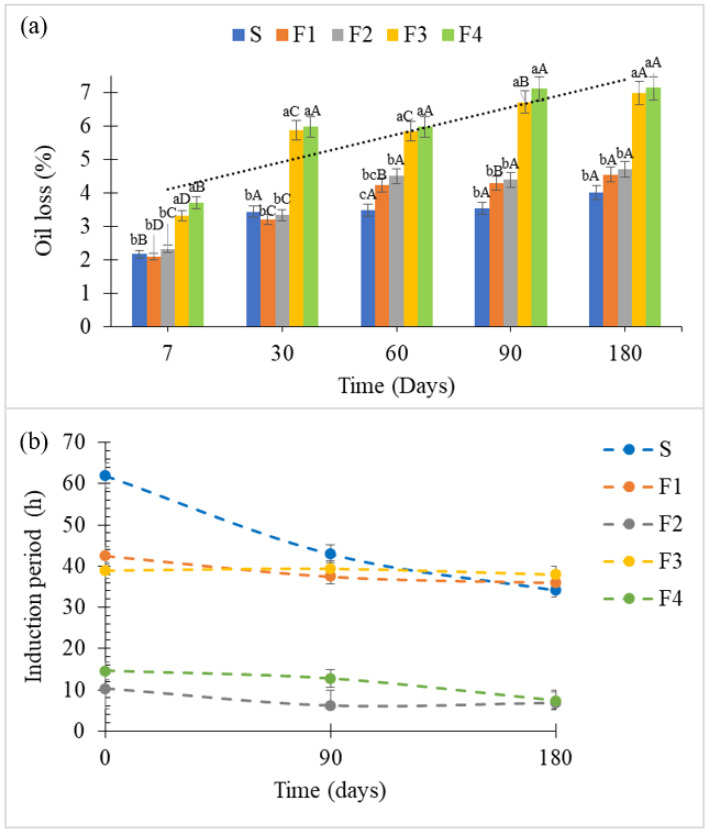
Oil loss (**a**) and Oxidative stability per induction period in hours (**b**). Note: In Figure 7a of the results marked with different letters showed significant differences (*p* ≤ 0.05). Lowercase letters indicate significant differences between samples, while uppercase letters denote significant differences over time. Formulations: F1 = 100% CS by mixer; F2 = 100% SL by mixer; F3 = 100% CS by ball mill; F4 = 100% SL by ball mill and S = Standard.

Oil loss (Figure 7a) was significantly linked to the production method. The ball mill process, which created a finer, more homogeneous structure (as seen in PSD), resulted in higher oil migration (up to 7.13% for F4). This suggests that excessive refining may weaken the fat network’s capacity to retain oil, aligning with the higher firmness observed in these samples, since migrated oil can contribute to matrix hardening. On the other hand, the mixer method promoted less particle breakdown, allowing reduced oil:matrix interaction, while aeration may have slowed oil migration (up to 4.7% to F1 and F2). No visible oil migration was observed on the cookie shells; however, a decrease in crispness was noted, particularly after 120 days of storage (Section 3.2.2). This indicates that, even without visible oil exudation, oil mobility within the filling can affect textural properties such as crispness.

According to [53] oil loss in fillings should preferably be absent or remain minimal, as the migration of liquid oils from the filling to the cookies can cause softening, loss of crispness, and other undesirable textural defects. To mitigate oil loss in ball-milled fillings, reducing milling time and/or adjusting stabilizing agents such as lecithin is recommended.

The lower oxidative stability of the SL-based fillings (F2 and F4), reflected in their shorter induction periods (Figure 7b), stems from their higher PUFA content combined with the absence of added antioxidants. The induction period indicates the time until significant lipid oxidation occurs, serving as a marker of oxidative stability. This reveals a formulation trade-off; although SL enhances the nutritional profile, its use requires antioxidant support to achieve adequate shelf life. In contrast, F1 and F3 showed similar and relatively stable induction periods, indicating effective oxidative protection. The Standard exhibited minimal variation over storage, confirming its stability. Both fillings contained ascorbyl palmitate, tocopherols, citric acid, and TBHQ (tert-butylhydroquinone). Additionally, fillings made with shortening had higher levels of saturated fatty acids, contributing to their superior oxidative stability compared to the SL-based systems.

Ma et al. [54] demonstrated that oxidative stability increases with saturated fatty acid content and decreases with PUFA levels, which are more prone to oxidation. Kumari et al. [55] demonstrated that the oxidative stability of omega-3-rich SLs is strongly dependent on effective antioxidant systems. The incorporation of rosemary extract significantly delayed oxidation and rancidity development under accelerated storage conditions (30 days), indicating that PUFA enrichment alone is insufficient to ensure oxidative stability compatible with commercial shelf life. Similar behavior has been reported for PUFA-enriched spreads; O’Dwyer et al. [56] observed accelerated oxidative deterioration and early off-flavor development in fish oil–based spreads, even in SL-based systems. Together, these findings support the present results, as the shorter induction periods observed for SL-based fillings suggest that, despite their nutritional advantages, additional oxidative protection is required to achieve adequate stability, particularly for long-term storage.

It is also noteworthy that the production methods (mixer or ball mill) had minimal negative impact on oxidative stability, suggesting that lipid composition and the presence of antioxidants were factors that were more decisive.

### 3.3. Sensory Evaluation

**Figure 8:**
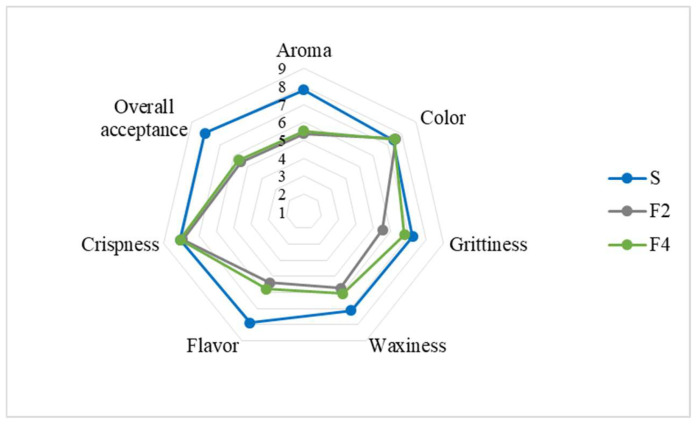
Sensory assessment of the strawberry sandwich-type cookies. Note: Nine-point hedonic scale: 1 = disliked extremely; 2 = disliked very much; 3 = disliked moderately; 4 = disliked slightly; 5 = neither liked nor disliked; 6 = liked slightly; 7 = liked moderately; 8 = liked very much; 9 = liked extremely. Formulations: F2 = 100% SL by mixer; F4 = 100% SL by ball mill and S = Standard.

The results of the sensory evaluation scores for the strawberry-filled cookies are presented in Figure 8. The filling color and cookie crispness attributes were well accepted, with no significant differences among samples, receiving mean scores between 7 and 8, corresponding to “liked moderately to liked very much” Pearson correlation analysis revealed a strong positive correlation (r ≥ 0.87) between the a* and C color parameters and the sensory color evaluation, suggesting that the more intense and red the filling color, the higher the visual acceptance by panelists. Cookies F2 and F4 received lower scores for grittiness, flavor, and overall acceptance (~5.8, corresponding to “neither liked nor disliked”; *p* < 0.05). A strong negative correlation (r = −0.99) was observed between sensory grittiness and D_50_, indicating that higher D_50_ values were associated with lower acceptance regarding grittiness. Cookie F2 exhibited the highest mean PSD (D_50_ ~185.2 μm) compared to the Standard and F4 fillings (D_50_ ≤ 86 μm), explaining its lower grittiness acceptance (5.5). Cookies F2 and F4 also showed low acceptance for aroma and waxiness attributes, with scores of 5 (“neither liked nor disliked”), and for flavor, scores of 6 (“slightly liked”; *p* > 0.05). Pearson correlation analysis indicated a strong negative correlation (r = −0.91) between oil loss and waxiness acceptance. Furthermore, cookies F2 and F4 had lower purchase intent scores, averaging 3 (“unsure whether I would buy this product”), in contrast to the Standard cookie, which scored ~1.7 (“would probably buy this product”; *p* < 0.05).

Regarding the acceptability index, which should be ≥70% [31] only the F2-filled cookie failed to reach the minimum acceptance value, scoring 68%. Consumers described it as having a granular texture with slight roughness (grittiness), and it was only positively rated for color. The F4-filled cookie had an acceptability index close to that of the Standard (≥70%) and was better accepted than F2, suggesting that the ball mill method favored sensory quality compared to the mixer method. Panelists described the Standard and F4 cookies as smooth, soft, and strawberry colored; however, F4 exhibited waxiness, likely due to higher oil loss.

To improve sensory acceptance, it is suggested to optimize refining time to reduce particle size and granularity, thereby minimizing grittiness. Residual waxiness may be reduced by the partial incorporation of structured lipids as the lipid base, as well as by replacing the flavor profile with aromas more compatible with peanut oil, such as chocolate or cocoa-based flavors.

## 4. Conclusions

This study demonstrated that palm oil-free SLs are a viable lipid base for sandwich cookie fillings, providing an improved nutritional profile with reduced saturated fat. Filling texture was affected by storage time, influencing product quality only after 90 days, while all filled cookies retained crispness for up to 120 days. Regarding sensory acceptance, SL-filled cookies exhibited some undesirable attributes, such as waxiness and grittiness, with the mixer-prepared filling (F2) showing lower acceptability (≤70%) than the others did. The main technological limitation was the lower oxidative stability of SL-based fillings due to their higher unsaturated fat content, indicating the need for optimized antioxidant systems. In addition, sensory performance requires the use of flavorings compatible with peanut oil to achieve better aroma integration (e.g., cocoa, chocolate, hazelnut, or almond). Overall, these formulations show potential for industrial scalability and represent a promising strategy for developing healthier lipid ingredients aligned with industrial and sustainability demands; however, further formulation adjustments are required to ensure optimal technological performance as lipid ingredients for the bakery and confectionery industry.

## Figures and Tables

**Table 1:** Fatty acid and triacylglycerol composition percentage (wt%).

Fatty Acids (%)		SL	CS
C12:0	Lauric	nd	0.75 ± 0.0
C14:0	Myristic	nd	1.01 ± 0.0
C16:0	Palmitic	7.32 ± 0.0	44.10 ± 1.2
C18:0	Stearic	10.77 ± 2.3	6.13 ± 0.5
C18:1	Oleic	34.50 ± 0.0	23.94 ± 0.8
C18:2	Linoleic	25.05 ± 0.2	21.42 ± 1.2
C18:3	Linolenic	1.53 ± 0.2	2.18 ± 0.1
C20:0	Arachidic	1.76 ± 0.2	0.47 ± 0.0
C22:0	Behenic	18.24 ± 0.4	nd
C24:0	Lignoceric	0.82 ± 2.0	nd
∑ Monounsaturated		34.50 ± 0.0	23.94 ± 0.8
∑ Polyunsaturated		26.58 ± 0.7	23.60 ± 2.0
∑ Saturated		38.92 ± 0.4	52.46 ± 1.7
∑ S_3_		2.28 ± 0.0	13.97 ± 0.0
∑ S_2_U		24.66 ± 0.0	39.68 ± 0.1
∑ SU_2_		46.20 ± 1.2	35.23 ± 0.1
∑ U_3_		26.87 ± 0.2	11.11 ± 0.5

Note: The values are expressed as mean ± SD (*n* = 3). nd = not detected. TAGS: S_3_ = tri-saturated; S_2_U = di-saturated–mono-unsaturated; SU_2_ = mono-saturated–di-unsaturated and U_3_ = tri-unsaturated.

**Table 2:** PSD parameters of strawberry fillings.

Fillings	PSD	0 Day	30 Days	90 Days	180 Days
F1	D_10_	56.4 ± 2.2 ^bcB^	45.0 ± 1.8 ^acA^	43.0 ± 1.8 ^bC^	40.7 ± 1.6 ^aC^
	D_50_	195.8 ± 2.8 ^cA^	156.0 ± 2.2 ^aC^	149.3 ± 2.2 ^bB^	140.9 ± 2.0 ^aD^
	D_90_	382.1 ± 4.4 ^acB^	304.2 ± 3.2 ^bC^	290.8 ± 3.1 ^aA^	274.5 ± 3.0 ^bD^
	D_4.3_	4.0 ± 0.9 ^bA^	3.2 ± 0.7 ^dB^	3.1 ± 0.7 ^cC^	3.0 ± 0.7 ^bC^
	Span	1.7 ± 0.4 ^bB^	1.3 ± 0.3 ^aA^	1.3 ± 0.3 ^aC^	1.3 ± 0.3 ^cD^
F2	D_10_	52.0 ± 3.5 ^bcC^	42.6 ± 2.9 ^acB^	40.5 ± 2.7 ^bA^	38.7 ± 2.5 ^aA^
	D_50_	185.2 ± 3.2 ^dC^	151.3 ± 2.6 ^aD^	143.8 ± 2.5 ^bA^	137.3 ± 2.4 ^aB^
	D_90_	392.5 ± 7.3 ^acB^	320.6 ± 6.0 ^cA^	304.8 ± 5.8 ^bC^	290.7 ± 5.5 ^cD^
	D_4.3_	4.1 ± 1.0 ^bB^	3.4 ± 0.8 ^cdC^	3.2 ± 0.8 ^cA^	3.1 ± 0.8 ^bA^
	Span	1.8 ± 0.8 ^bB^	1.5 ± 0.6 ^aC^	1.4 ± 0.6 ^abA^	1.4 ± 0.6 ^cA^
F3	D_10_	32.9 ± 2.5 ^aB^	25.2 ± 1.9 ^bA^	25.3 ± 1.9 ^aA^	24.3 ± 1.8 ^cA^
	D_50_	115.9 ± 6.6 ^bC^	88.5 ± 5.0 ^cA^	88.7 ± 5.0 ^aA^	85.5 ± 4.9 ^bB^
	D_90_	497.4 ± 7.8 ^bB^	379.0 ± 5.9 ^dC^	379.9 ± 5.9 ^cC^	365.7 ± 5.7 ^aD^
	D_4.3_	7.6 ± 1.0 ^aC^	5.9 ± 1.7 ^aB^	5.9 ± 1.7 ^bB^	5.7 ± 1.7 ^cA^
	Span	4.0 ± 0.9 ^aB^	3.1 ± 0.7 ^cA^	3.1 ± 0.7 ^cA^	3.0 ± 0.7 ^aC^
F4	D_10_	16.5 ± 0.7 ^deC^	14.0 ± 0.6 ^dA^	13.0 ± 0.6 ^cB^	11.9 ± 0.5 ^dD^
	D_50_	80.0 ± 0.3 ^aB^	67.7 ± 0.3 ^bA^	63.2 ± 0.3 ^dAC^	57.7 ± 0.3 ^cC^
	D_90_	366.9 ± 3.0 ^acA^	310.2 ± 2.5 ^cB^	289.8 ± 2.4 ^aE^	264.6 ± 2.3 ^bC^
	D_4.3_	1.3 ± 0.3 ^cB^	1.1 ± 0.3 ^bA^	1.0 ± 0.2 ^aAC^	1.0 ± 0.2 ^dC^
	Span	4.0 ± 1.8 ^aC^	3.4 ± 1.5 ^dB^	3.2 ± 1.4 ^cA^	2.9 ± 1.4 ^aD^
Standard	D_10_	14.1 ± 0.8 ^deB^	14.1 ± 0.8 ^dB^	13.5 ± 0.8 ^cA^	13.5 ± 0.8 ^bA^
	D_50_	86.3 ± 3.0 ^aA^	86.1 ± 3.0 ^cA^	83.2 ± 3.0 ^acB^	83.9 ± 3.0 ^bB^
	D_90_	313.8 ± 6.2 ^dB^	313.2 ± 6.2 ^acB^	302.3 ± 6.0 ^bA^	304.9 ± 6.0 ^dA^
	D_4.3_	1.2 ± 0.2 ^cC^	1.2 ± 0.2 ^bC^	1.1 ± 0.2 ^aAB^	1.1 ± 0.2 ^aA^
	Span	3.5 ± 1.6 ^aB^	3.5 ± 1.6 ^dB^	3.3 ± 1.5 ^cC^	3.4 ± 1.5 ^bC^

Note: Values are expressed as mean ± SD (*n* = 5). Results marked with different letters indicate significant differences (*p* ≤ 0.05). Lowercase letters indicate significant differences between samples, whereas uppercase letters denote significant differences over time. Formulations: F1 = 100% CS by mixer; F2 = 100% SL by mixer; F3 = 100% CS by ball mill; F4 = 100% SL by ball mill.

**Table 3:** Color characterization of strawberry fillings.

Fillings	Color	0 Day	30 Days	90 Days	180 Days
F1	L	64.8 ± 0.4 ^aB^	63.0 ± 0.2 ^aCD^	61.9 ± 0.1 ^bDE^	67.5 ± 0.5 ^aA^
	a*	30.5 ± 0.5 ^bE^	31.9 ± 0.2 ^aD^	30.6 ± 0.1 ^bE^	38.4 ± 0.3 ^aA^
	b*	12.4 ± 0.2 ^bC^	12.5 ± 0.1 ^abC^	11.8 ± 0.1 ^aD^	15.5 ± 0.1 ^aA^
	h°	0.39 ± 0.0 ^bB^	0.37 ± 0.0 ^aDE^	0.37 ± 0.0 ^bE^	0.38 ± 0.0 ^bC^
	C	32.9 ± 0.5 ^bE^	34.2 ± 0.2 ^aD^	32.8 ± 0.2 ^cE^	41.4 ± 0.3 ^aA^
F2	L	65.2 ± 0.3 ^aA^	60.4 ± 0.3 ^bB^	57.6 ± 0.2 ^dD^	57.5 ± 0.6 ^dD^
	a*	27.7 ± 0.2 ^dD^	30.2 ± 0.7 ^bC^	31.0 ± 0.2 ^bBC^	35.5 ± 0.5 ^bA^
	b*	11.7 ± 0.1 ^cD^	12.1 ± 0.4 ^aCD^	12.6 ± 0.0 ^abC^	14.0 ± 0.3 ^bB^
	h°	0.40 ± 0.0 ^aA^	0.38 ± 0.0 ^aB^	0.39 ± 0.0 ^aB^	0.38 ± 0.0 ^bB^
	C	30.1 ± 0.3 ^cD^	32.6 ± 0.8 ^bC^	33.5 ± 0.2 ^bBC^	38.2 ± 0.6 ^bA^
F3	L	58.1 ± 0.3 ^bD^	59.7 ± 0.0 ^bD^	57.9 ± 0.1 ^cD^	64.6 ± 1.8 ^bA^
	a*	33.2 ± 0.4 ^aB^	33.1 ± 0.3 ^aB^	33.1 ± 0.4 ^aB^	38.3 ± 1.2 ^aA^
	b*	13.8 ± 0.1 ^aB^	12.3 ± 0.2 ^abC^	12.1 ± 0.2 ^bcC^	15.4 ± 0.5 ^aA^
	h°	0.39 ± 0.0 ^abB^	0.36 ± 0.0 ^bD^	0.35 ± 0.0 ^cD^	0.31 ± 0.0 ^bE^
	C	36.0 ± 0.4 ^aCD^	35.3 ± 0.3 ^aD^	35.2 ± 0.4 ^aD^	41.2 ± 1.3 ^aB^
F4	L	57.3 ± 0.7 ^bC^	58.0 ± 0.9 ^cBC^	57.9 ± 0.1 ^cBC^	62.2 ± 1.1 ^cA^
	a*	28.7 ± 0.9 ^cC^	30.0 ± 1.1 ^bC^	29.2 ± 0.1 ^cB^	34.1 ± 0.5 ^bA^
	b*	11.8 ± 0.6 ^cB^	11.0 ± 0.5 ^bC^	10.3 ± 0.0 ^cD^	13.2 ± 0.2 ^cA^
	h°	0.39 ± 0.1 ^bA^	0.35 ± 0.0 ^bE^	0.34 ± 0.0 ^dC^	0.37 ± 0.0 ^cB^
	C	31.1 ± 1.1 ^cC^	32.0 ± 1.2 ^bC^	31.0 ± 0.1 ^dC^	36.6 ± 0.5 ^cA^
Standard	L	64.7 ± 0.5 ^aBC^	64.1 ± 0.7 ^aC^	68.08 ± 0.14 ^aA^	67.9 ± 0.9 ^aA^
	a*	20.8 ± 0.3 ^eC^	20.8 ± 0.6 ^cBC^	19.61 ± 0.2 ^dD^	24.5 ± 0.6 ^cA^
	b*	14.7 ± 0.1 ^dCD^	14.3 ± 0.5 ^cDE^	14.04 ± 0.24 ^dE^	16.4 ± 0.2 ^dA^
	h°	0.40 ± 0.1 ^abA^	0.38 ± 0.1 ^aBC^	0.39 ± 0.13 ^aAB^	0.40 ± 0.0 ^aA^
	C	22.5 ± 0.3 ^dCD^	22.4 ± 0.7 ^cCD^	19.5 ± 3.2 ^eE^	26.6 ± 0.6 ^dA^

Note: Values are expressed as mean ± SD (*n* = 5). Results marked with different letters indicate significant differences (*p* ≤ 0.05). Lowercase letters indicate significant differences between samples, whereas uppercase letters denote significant differences over time. Formulations: F1 = 100% CS by mixer; F2 = 100% SL by mixer; F3 = 100% CS by ball mill; F4 = 100% SL by ball mill.

**Table 4:** Water activity values of filled cookies.

Fillings	0 Day	30 Days	90 Days	180 Days
F1	0.582 ± 0.2 ^bA^	0.362 ± 0.1 ^bcC^	0.368 ± 0.1 ^aC^	0.511 ± 0.3 ^aB^
F2	0.452 ± 0.1 ^dB^	0.461 ± 0.1 ^abAB^	0.357 ± 0.2 ^aC^	0.518 ± 0.2 ^aAB^
F3	0.517 ± 0.1 ^cA^	0.398 ± 0.5 ^bcBCD^	0.384 ± 0.1 ^aCD^	0.458 ± 0.1 ^aAB^
F4	0.555 ± 0.1 ^dABC^	0.462 ± 0.2 ^aA^	0.363 ± 0.1 ^aC^	0.507 ± 0.2 ^aABC^
Standard	0.581 ± 0.1 ^aA^	0.317 ± 0.1 ^cD^	0.359 ± 0.2 ^bD^	0.485 ± 0.9 ^aB^
**Shells**				
F1	0.313 ± 0.1 ^aD^	0.309 ± 0.0 ^bcD^	0.346 ± 0.2 ^bC^	0.446 ± 0.2 ^aA^
F2	0.313 ± 0.1 ^aD^	0.307 ± 0.0 ^cDE^	0.311 ± 0.0 ^dDE^	0.424 ± 0.5 ^cdB^
F3	0.313 ± 0.1 ^aC^	0.326 ± 0.0 ^bC^	0.378 ± 03 ^aB^	0.443 ± 0.7 ^dB^
F4	0.313 ± 0.1 ^aE^	0.401 ± 0.5 ^aB^	0.326 ± 0.0 ^cdDE^	0.449 ± 0.8 ^cB^
Standard	0.313 ± 0.1 ^aD^	0.324 ± 0.1 ^bcCD^	0.334 ± 0.0 ^bcC^	0.417 ± 0.5 ^bA^

Note: Values are expressed as mean ± SD (*n* = 3). Results marked with different letters indicate significant differences (*p* ≤ 0.05). Lowercase letters indicate significant differences between samples, whereas uppercase letters denote significant differences over time. Formulations: F1 = 100% CS by mixer; F2 = 100% SL by mixer; F3 = 100% CS by ball mill; F4 = 100% SL by ball mill.

## Data Availability

The original contributions presented in this study are included in the article. Further inquiries can be directed to the corresponding author.

## Abbreviations

The following abbreviations are used in this manuscript: *a*_w_—Water activity; BOPP—Biaxially oriented polypropylene; OLL—oleic–linoleic–linoleic; LLL—linoleic–linoleic–linoleic; StLBe—stearic–linoleic–behenic; BeBeBe—behenic–behenic–behenic; POP—palmitic–oleic–palmitic; PLP—palmitic–linoleic–palmitic; POO—palmitic–oleic–oleic; PLO—palmitic–linoleic–oleic; PLL—palmitic–linoleic–linoleic; PPP—palmitic–palmitic–palmitic; CN—Carbon number; CS—Commercial shortening; DAGs—Diacylglycerols; DSC—Differential scanning calorimetry; FAC—Fatty acid composition; GC—Gas chromatography; HFCO—Fully hydrogenated crambe oil hard fat; INPI—National Institute of Industrial Property; MUFAs and PUFAs—Mono- and polyunsaturated fatty acids; NMR—Nuclear magnetic resonance; MCPD—Monochloropropane-1,2-diol; PO—High-oleic peanut oil; PSD—Particle size distribution; SFC—Solid fat content; SL—Structured lipid; SO—Soybean oil; TAG—Triacylglycerol; TBHQ—Tert-butylhydroquinone and THF—Tetrahydrofuran.
